# The Pathology of the Teeth

**Published:** 1874-01

**Authors:** 


### BOOK REVIEWS.

*The Pathology of the Teeth, with Special Reference to their Anatomy and Physiology*: By CARL WEDL, M. D. Professor of Histology in the University of Vienna, etc.; Translated from the German by W. E. BOARDMAN, M. D.; With Notes by T. B. HITCHCOCK, M. D., D, M. D., Professor of Dental Pathology and Therapeutics in Harvard University, etc. With 105 illustrations. Phila.: Lindsay and Blakiston, 1872.

In this volume of 443 pages, we have a work with which no dentist can afford to be unacquainted. It is written by a distinguished German scholar and investigator, whose name alone must guarantee its excellence. Nearly twenty years ago Prof. Wedl published a work on pathological histology which has made him widely and favorably known to the scientific world in Germany, and measurably so in England and this country. Being an old observer and laborer in the fields of pathology, he was peculiarly fitted to investigate the pathology of the teeth and to bring together the scattered results of such investigations as have been made by others. And this he has accomplished in a manner so masterly that he must call out the gratitude of the whole profession.

In the first portion of the work, seventy-five pages are devoted to dental anatomy and physiology. The normal state of the teeth and contiguous parts is lucidly and with sufficient minuteness set before the reader. This was necessary as a point of departure for the consideration of those aberrations from the normal state which form the proper subject of a work on pathology.

Coming to the second part of the volume, the author has divided the different varieties of morbid processes into seven classes, as follows:

I. Irregularities in the Formation of the Teeth,
II. Inflammations,
III. Atrophies,
IV. Hypertrophies,
V. New Formations,
VI. Anomalies of the Secretions,
VII. Neuroses.

Each of these morbid affections is traced in all the various tissues with which the dentist has to deal, and that with great minuteness of detail and subtlety of research. Here is the author's plan of treating the teeth and their adnexa with reference to their morbid states. Under these heads he has grouped such important facts and researches as have appeared in the society reports and publications of all countries, and which were of course inaccessible, except in the merest fragments, to the mass of practioners. To have barely collected these scattered facts and investigations would have been an acceptable work. This, however, is only the fraction of what has been done. Professor Wedl has himself made extensive and most valuable additions from his own direct researches. While acting as a seine to protect his readers from divers errors and false observations, he has made large contributions, by means of Heidel's manuscripts and his own observations, of matter not before made public.

The reader will have reason to admire the numerous engravings with which the work is profusely illustrated. They are from the same cuts that were used in the German edition, and are of unusual excellence.

A constant reference is made throughout the volume to the "Atlas of the Pathology of the Teeth." This consists of a collection of 145 engravings of malformations and diseases of the teeth, with explanations of the same in German and English. It is a very desirable accompaniment to the book, which is nevertheless complete without it, and has abundant illustrations of its own.

After a careful examination of this most excellent work by Prof. Wedl, I am convinced that every dentist who desires to keep abreast with his time will read it. It is not a light production, to be disposed of in a rapid perusal, but is fitted to be the companion of him who would pursue his investigations into the mysterious phenomena of disease, while conducting his daily practice. It does not describe methods of operating, and may in this particular disappoint the student, who is too apt to give his whole force to the acquisition of skill in performing operations, while proportionately neglecting to thoroughly understand pathological conditions.

There can be no doubt that this work will contribute largely to the study and advancement of dental science in this country. It has many faults; some of style, and some in the subject-matter itself: but it has great excellences. If some of its topics are treated with a disappointing meagerness, others are presented with a fulness and originality that are both surprising and gratifying.

It has been remarked, in a manner that has come to my knowledge, that this work by Prof. Wedl is too elaborate, too recondite, too profound, too difficult, for the average dental student. So much the worse for the average dental student! I do not want to see works written down to the level of the average dental student. Let him come up to the high-water mark attained in our best works.

